# Opportunities for using the USCDM for impact

**DOI:** 10.1002/alz70860_101359

**Published:** 2025-12-23

**Authors:** Bryan Tysinger, Duncan Ermini Leaf, Geoffrey Joyce, Johanna Thunell, Hanke Heun‐Johnson, Mireille Jacobson, Sidra Haye, Manying Cui, Julie M Zissimopoulos

**Affiliations:** ^1^ University of Southern California, Los Angeles, CA, USA

## Abstract

**Background:**

The United States Cost of Dementia Model (USCDM) is a dynamic microsimulation model that generates nationally‐representative estimates of comprehensive cost of dementia of individuals, families, payers and society. The model assesses cost across different stakeholders, and quantifies the impact on costs of changes in treatments, care, policies and population health for different populations. USCDM provides valuable insights for the research community, however its computational complexity may limit its full impact potential.

**Method:**

To increase access to broad audiences, the authors developed a prototype emulator that replicates the behavior of the full USCDM in a more computationally efficient and user friendly manner. The emulator used linear regression to predict key model outputs: number of people with any ADL limitations, the number of people living in nursing homes, diabetes prevalence, heart disease prevalence, dementia prevalence, Medicare costs, Medicaid costs, out‐of‐pocket medical costs, and total costs for all payers. We assessed impact on these outcomes from a reduction in all‐cause mortality incidence, reduction in ADL limitations, and a delay in dementia onset. We compared regression model predictions to the actual USCDM output in terms of accuracy, interpretability, and efficiency. We performed these comparisons using a test data set of USCDM outputs that were not part of the sample data set used to estimate the emulator regression model.

**Result:**

For scenarios representing a change in all‐cause mortality incidence, a reduction in the number of ADL limitations, or delayed dementia onset, we assessed accuracy by relative error – the percent difference between each USCDM output and the emulator prediction – and found the emulator outcome results were within 0.01% of USCDM outputs across all demographic population we assessed. The emulator yields these predictions in under 10 seconds while the USCDM would take 23 minutes in a 20‐processor parallel computing environment.

**Conclusion:**

By improving accessibility to USCDM outputs, the emulator enhances usability for researchers conducting health economics analyses, policymakers evaluating the impact of interventions, and the layperson seeking a clearer understanding of dementia‐related health and economic outcomes.

